# Clinical Assessment of Obesity Beyond Body Mass Index: Adiposity Evaluation, Body-Composition Phenotyping and Disease Staging: A Narrative Review

**DOI:** 10.3390/diagnostics16172837

**Published:** 2026-09-03

**Authors:** Ion-Vladut Udroiu, Alin Albai, Sandra Lazar, Adina Braha, Laura Gaita, Bogdan Timar, Alexandra Sima

**Affiliations:** 1 Doctoral School of Medicine, “Victor Babes” University of Medicine and Pharmacy, 300041 Timisoara, Romania; ion-vladut.udroiu@umft.ro; 2 Department of Diabetes, “Pius Brinzeu” Emergency Hospital, 300723 Timisoara, Romania; albai.alin@umft.ro (A.A.); braha.adina@umft.ro (A.B.); bogdan.timar@umft.ro (B.T.); sima.alexandra@umft.ro (A.S.); 3 Centre for Molecular Research in Nephrology and Vascular Disease, “Victor Babes” University of Medicine and Pharmacy, 300041 Timisoara, Romania; 4 Second Department of Internal Medicine, “Victor Babes” University of Medicine and Pharmacy, 300041 Timisoara, Romania; 5 First Department of Internal Medicine, “Victor Babes” University of Medicine and Pharmacy, 300041 Timisoara, Romania; 6 Department of Hematology, Emergency Municipal Hospital, 300254 Timisoara, Romania

**Keywords:** obesity assessment, body mass index, anthropometry, body composition, bioelectrical impedance analysis

## Abstract

Obesity is usually classified or assessed using the body mass index (BMI), mainly because BMI is simple and easy to apply. Its simplicity is also its main weakness. BMI does not measure fat mass, fat-free mass, or fat distribution. This is a relevant limitation, since individuals with the same BMI may have different amounts of visceral adipose tissue and different cardiometabolic risks. Recent recommendations from the Commission on Clinical Obesity also emphasize that BMI should be used mainly as a screening tool, while excess adiposity should be confirmed using additional anthropometric or body composition measures. This narrative review examines clinical methods for obesity assessment beyond BMI and discusses their practical value in routine care. A literature search was performed in PubMed and Google Scholar for articles published up to August 2026. Guidelines, consensus statements, reviews, meta-analyses, and original studies were considered. The review focused on anthropometric indices, body composition techniques, and imaging-based methods used to assess adiposity, fat distribution, and obesity-related risk. The reviewed evidence supports a complementary approach. Anthropometric indices help evaluate abdominal adiposity and cardiometabolic risk. Body composition methods add information on fat mass, fat-free mass, and regional distribution. Imaging techniques offer the most detailed assessment of visceral, subcutaneous, and ectopic fat, although their routine use is limited by cost, availability, technical requirements, or radiation exposure. BMI remains useful for initial screening, but it is not sufficient for a complete obesity assessment. Combining anthropometric, body composition, and imaging-based methods may improve the evaluation of excess adiposity, fat distribution, and clinical risk.

## 1. Introduction

Since 1990, global obesity rates have more than doubled. In 2022, obesity affected one in eight people worldwide, with an estimated 890 million adults (aged 20 years and over) living with the condition. According to the World Obesity Atlas (2023), this number is expected to reach nearly 1.5 billion by 2035 [1]. This is important considering that obesity is a major risk factor for numerous other conditions, including type 2 diabetes mellitus (T2DM), metabolic dysfunction-associated steatotic liver disease, osteoarthritis, various types of cancer, depression and related mental health disorders, and cardiovascular diseases (CVD) like hypertension, atrial fibrillation, stroke, myocardial infarction and heart failure [2,3,4].

Conventionally, obesity is diagnosed based on body mass index (BMI), which is calculated as the ratio of body weight in kilograms divided by height in square meters (kg/m^2^). Four BMI categories are proposed by the World Health Organization (WHO) and are widely utilized to estimate an individual’s weight status: underweight (<18.5 kg/m^2^), normal weight (18.5–24.9 kg/m^2^), overweight (25.0–29.9 kg/m^2^), and obesity (≥30.0 kg/m^2^) [5]. Because obesity is a heterogeneous condition, it cannot be fully assessed through BMI alone. This measure has important limitations and may lead to misleading interpretations. It does not differentiate between fat mass (FM) and fat-free mass (FFM), nor does it reflect fat distribution [6,7].

Adipose tissue is not distributed uniformly throughout the body. The metabolic impact of this tissue does not depend only on the amount, but also on its distribution [8,9]. Two major abdominal adipose compartments are usually described: subcutaneous adipose tissue (SAT) and visceral adipose tissue (VAT) [10,11]. SAT has different physiological roles, but mainly acts as an energy storage depot [8,9,12]. VAT is consistently associated with insulin resistance, metabolic syndrome, T2DM, CVD, and increased mortality, even if it represents a smaller proportion of total body fat [8,10,11]. Multiple studies reported a stronger association between VAT and metabolic abnormalities in individuals who are overweight or obese compared with SAT [10,13,14]. These differences may explain why individuals with similar body weight or BMI can have very different cardiometabolic risk profiles. Therefore, accurate and clinically practical methods for assessing VAT are important in obesity assessment [8,11].

Recent expert consensus statements from the Commission on Clinical Obesity redefined obesity and its diagnostic approach. The Commission’s recommendations emphasize that excess adiposity needs confirmation from other anthropometric or body composition measures, in addition to BMI, which could be used only for screening [15,16]. This broader approach is consistent with other recent frameworks that incorporate abdominal adiposity, body composition, obesity-related complications, and functional impairment into diagnosis and disease staging [17,18]. This change in diagnosis highlights the need for practical, accessible, and reliable methods to assess adiposity in clinical practice.

This recent change in perspective forms the basis of the present review, which aims to explore and compare clinical methods for obesity assessment beyond BMI, including anthropometric measurements, body composition techniques, and imaging methods. Several recent reviews have summarized the expanding range of tools available for obesity assessment [19,20]. These include conventional anthropometric measures, but also a growing number of body composition and imaging techniques. The present review builds on this literature but focuses more specifically on how these methods fit within the newer diagnostic approach to obesity. We consider what each method can add in terms of adiposity, fat distribution, and body composition, as well as its practical use in clinical settings. The objective is to evaluate these approaches in terms of clinical utility, feasibility, strengths, and limitations, and to clarify how they may complement BMI in routine clinical practice.

## 2. Methods

This narrative review was based on a literature search focused on clinical methods used for obesity assessment beyond BMI, including articles published up to August 2026. Relevant publications were identified through PubMed and Google Scholar. The search included international guidelines, consensus statements, narrative and systematic reviews, meta-analyses, and original studies addressing anthropometric indices, body composition methods, and imaging-based assessment of adiposity.

The main search terms included “obesity assessment”, “body mass index”, “BMI limitations”, “waist circumference”, “waist-to-height ratio”, “waist-to-hip ratio”, “body composition”, “bioelectrical impedance analysis”, “dual-energy X-ray absorptiometry”, “computed tomography”, “magnetic resonance imaging”, “ultrasound”, “visceral adipose tissue”, “sarcopenic obesity”, and “digital anthropometry”, used alone or in combination. Additional relevant articles were identified by screening the reference lists of selected publications.

Priority was given to clinically relevant studies and recent publications, especially those evaluating the applicability, strengths, limitations, reproducibility, and diagnostic performance of different methods. Older landmark articles were also included when relevant for historical context or for explaining the development of specific obesity assessment tools. Because this was designed as a narrative review, no formal systematic review protocol, risk of bias assessment, or meta-analysis was performed. This narrative review was based exclusively on previously published literature. It did not involve the collection or analysis of individual-level participant data. Ethical approval and informed consent were not required.

## 3. Body Mass Index: Clinical Utility and Limitations

### 3.1. Historical Background

The concept of the BMI has its roots in the 19th century, when Belgian statistician Adolphe Quetelet developed what he called the “Quetelet Index” to describe the characteristics of a “normal” man [21,22]. However, his goal was not to study obesity. Nearly a century later, Ancel Keys (1904–2004) emphasized the importance of body weight relative to height. In his 1972 paper titled “Indices of Relative Weight and Obesity,” he validated the Quetelet Index and introduced the term “Body Mass Index” [23].

### 3.2. Clinical Utility of BMI

Today, BMI is one of the most widely used tools for classifying individuals based on weight status. It is a standardized and globally recognized metric, with a BMI over 30 kg/m^2^ used as the threshold for defining obesity in various health guidelines [5,24,25,26]. The main advantages of BMI are its simplicity, low cost, and ease of application. It is calculated only from weight and height (kg/m^2^), which makes it quick to apply with minimal effort. It does not require radiation, imaging, complex assessments, or even a medical appointment, making it very accessible for medical providers. Also, individuals can calculate their own BMI to track their health status related to weight [27].

Because it is simple and inexpensive, BMI is frequently used in research, particularly in epidemiological studies involving large populations. It is commonly used to examine associations between body weight and different health outcomes. These data have contributed to the development of public health strategies focused on obesity and its complications. The correlation between BMI and mortality can be used to estimate the impact on public health of obesity. Numerous large-scale population studies have demonstrated a J-shaped relationship between BMI and the risks of morbidity and mortality, suggesting that a BMI above 25 kg/m^2^ is associated with a significant increase in health risks and mortality rates [28,29,30]. It is important to note that the association between BMI and mortality can be influenced by factors like smoking or chronic diseases. These may lead to reverse causality, as these factors can themselves influence a person’s BMI [31,32]. To reduce these biases, Di Angelantonio et al. (2016) conducted a large meta-analysis including over 10 million adults from 239 studies [30]. The analysis was restricted to approximately 4 million individuals who were never-smokers, free of chronic diseases at the start, and followed for at least five years. These exclusions significantly reduced potential confounding and confirmed that BMI over 25 kg/m^2^ is associated with increased risk of all-cause mortality. This is further confirmed by another meta-analysis of 15 randomized clinical trials with 17,186 participants conducted by Kritchevsky et al. (2015), which concluded that individuals who achieved intentional weight loss had a 15% lower risk of all-cause mortality [33].

Even if BMI itself does not directly cause mortality, the higher mortality risk seen with increasing BMI is explained by its relationship with major chronic diseases such as T2DM, hypertension, CVD, and several types of cancers [2,3,34,35]. While these associations are well documented in observational studies, genetic approaches such as Mendelian randomization (MR) provide an alternative to test if these links are truly causal [36]. They are less affected by confounding or reverse causation. Nordestgaard et al. (2012) conducted an MR analysis and reported that every 4 kg/m^2^ increase in BMI raised the risk of ischemic heart disease by 52% [37]. More recently, Wu et al. (2025) used an MR phenome-wide association study (PheWAS) and found causal links between BMI and several chronic diseases like CVD, T2DM and chronic renal failure [38]. Recently, over the last few years, several studies have shown that a higher BMI increases the risk of severe outcomes from SARS-CoV-2 infection (COVID-19 disease). It increases the risk of mortality in hospitalized infected patients and the need for mechanical ventilation [39,40,41].

Despite these advantages, considering the complexity of obesity, BMI does not provide the complete picture of the disease. It may even sometimes misclassify individuals. But because of its strengths, especially low cost, minimal effort, and speed, it will likely remain the most widely used tool for assessing obesity, even when its limitations are well recognized.

### 3.3. Limitations of BMI

BMI has some well-documented disadvantages, which limit its clinical utility and highlight the need for additional tools to better evaluate obesity.

#### 3.3.1. Poor Correlation with Body Composition

Excess body fat is a defining characteristic of obesity, but BMI does not measure it directly [15]. Studies show only a modest correlation between BMI and body fat percentage [7,24,42,43,44]. Gallagher et al. (1996) showed that BMI explained only about 25% of the variance in body fat among 706 men and women [7]. Smalley et al. (1990) evaluated body fat with densiometric analysis and found that the 95% confidence intervals for body fat percentage at a given BMI were very wide [43]. They found that for a man with BMI of 27, the body fat percentage would be as little as 10% or as much as 31.7%. This range goes from near malnutrition to clear obesity. Another study done by Romero-Corral et al. (2008) using Third National Health and Nutrition Examination Survey (NHANES) reported similar findings [44]. In people with a BMI of 25 kg/m^2^, body fat percentage evaluated by Bioelectrical Impedance Analysis (BIA) varied from 14% to 35% in men and from 26% to 43% in women. These results show that people classified as “normal weight” by BMI may in fact have obesity. Overall, BMI has limited diagnostic accuracy, especially between 25 and 30 kg/m^2^ [44]. This fact suggests that the prevalence of obesity may be underestimated.

#### 3.3.2. Failure to Reflect Fat Distribution

Another important limitation of BMI is that it does not show how fat is distributed in the body. Yet, the way fat is distributed in the body has major implications for health. Jean Vague, professor at the Faculty of Medicine in Marseille, emphasized this in his papers from 1940 and 1956 [45,46]. He described the differences between “android” obesity and “gynoid” obesity. Android obesity, with fat mainly in the upper body, was linked with metabolic disturbances, premature CVD, and T2DM. Gynoid obesity, with fat concentrated in the lower body, was associated more with mechanical complications [45,46].

Following studies showed that VAT plays a central role in the metabolic complications of abdominal obesity [8,10,11,13,47]. VAT is strongly associated with an unfavorable metabolic risk profile and predicts a higher risk of developing T2DM and CVD. Individuals with high VAT, even without clinical obesity, are the subgroup most affected by severe insulin resistance [8,10,13,47].

BMI cannot evaluate VAT, and two individuals with the same BMI can have very different distributions of SAT and VAT, resulting in very different cardiometabolic risks [42,48]. As a result, BMI often underestimates risk in people with excess VAT but normal BMI, and overestimates it in those with predominantly SAT. Simple anthropometric indices that reflect abdominal adiposity more directly, such as waist circumference (WC) or waist-to-hip ratio (WHR), provide better estimates of risk and will be discussed later in this review [24,42,48].

This limitation shows that BMI alone is not enough to evaluate cardiometabolic risk.

#### 3.3.3. Misleading Classification of Individuals

BMI often misclassifies people because it does not separate body types or metabolic profiles. Individuals with high muscle mass but little FM, such as athletes, may be wrongly classified as overweight or even obese [49]. With aging, the balance of muscle and fat changes and leads to new phenotypes that BMI cannot capture. One important phenotype is sarcopenic obesity, when individuals have low muscle mass combined with high body FM [48,50]. The coexistence of obesity and sarcopenia is associated with frailty, higher risk of metabolic disorders such as insulin resistance, and increased mortality [51,52]. Because BMI does not evaluate FM or muscle mass, individuals with sarcopenic obesity may have normal BMI, even though their health risks are much higher [48].

Another phenotype that BMI fails to identify is often called TOFI, meaning “thin outside, fat inside.” Individuals with this phenotype have low SAT but excess VAT. A normal BMI can hide the fact that they have a higher cardiometabolic risk. This profile may be present in one quarter of normal-weight individuals [48]. At the other end of the spectrum, some people classified as obese by BMI have a more favorable metabolic profile. This condition is known as metabolically healthy obesity (MHO) [48]. Stefan et al. (2008) [53] examined this phenotype using Magnetic Resonance Imaging (MRI) and MR spectroscopy to measure total FM, SAT, and VAT. They found that a subgroup of obese individuals had insulin sensitivity and carotid intima-media thickness comparable to normal-weight subjects [53]. In another study, Wildman et al. (2008) [54] evaluated a 5440 U.S. adults sample from NHANES 1999–2004 and reported that 31.7% of obese individuals (classified by BMI) were metabolically healthy. In this study, metabolic health was defined as having maximum one abnormality, such as elevated triglycerides, high C-reactive protein, high blood pressure, elevated fasting plasma glucose, insulin resistance or low high-density lipoprotein cholesterol level [54]. These two studies are only part of the evidence, but they underline the fact that BMI cannot separate metabolically healthy from unhealthy obesity.

#### 3.3.4. Population-Specific Cut-Off Points

Although we presented WHO cut-off points for BMI in the Introduction, they were derived mainly from Western populations and may not apply equally to all ethnic groups [5]. In Asian populations, lower thresholds are more appropriate. For example, Zhou (2002) conducted a study on the relationship between BMI, all-cause mortality, and CVD in China and suggested cut-offs of ≤18.5 for underweight, 24–27.9 for overweight, and ≥28 kg/m^2^ for obesity in Chinese adults [55]. Similarly, the Indian Consensus Group recommended overweight at ≥23 kg/m^2^ and obesity at ≥25 kg/m^2^ for Asian Indians. These thresholds were adopted by the National Institute of Health and Care Excellence in the UK and later by the American Diabetes Association for Asian populations in the United States [56]. Evidence shows that many Asians develop T2DM and cardiovascular complications at BMI levels below 25 kg/m^2^. At the same BMI, they usually have a higher percentage of FM compared to European populations. On the other hand, some Pacific populations show the opposite pattern, with lower FM for the same BMI [57]. These differences underline the fact that a universal BMI classification does not reflect risk equally across populations. Specific ethnic cut-offs may provide better guidance in clinical practice.

These limitations highlight the need for complementary methods that can better characterize excess adiposity, in line with recent recommendations from the Commission on Clinical Obesity [15]. The following sections will explore these approaches.

## 4. Anthropometric Measures Beyond BMI

### 4.1. Waist Circumference

WC is one of the simplest methods used for assessing abdominal adiposity. It is particularly useful in individuals with a BMI below 35 kg/m^2^; otherwise, it adds limited additional predictive value beyond BMI [58].

According to the WHO protocol, this metric should be measured at the midpoint between the top of the iliac crest and the lower margin of the last palpable rib [59]. Different cut-off points are used around the globe to define increased cardiometabolic risk and metabolic syndrome. The most widely used thresholds, supported by multiple organizations including the International Diabetes Federation (IDF), American Heart Association (AHA) and WHO, define two levels of risk in Caucasian populations [26,58,59,60,61]. A WC ≥ 94 cm in men and ≥80 cm in women indicates increased risk, while WC ≥ 102 cm in men and WC ≥ 88 cm in women indicate substantially higher risk [59]. These lower thresholds are also applied for the Sub-Saharan, the Middle East, and the Eastern Mediterranean populations. The higher thresholds are especially used in United States, Canada and Europe [26,59,60]. However, these cut-offs are not universal. Population-specific studies show that different thresholds may be more appropriate in other ethnic groups. In Asian populations, lower values are recommended. Although some variation exists between countries, mostly the cut-offs are around ≥90 cm for men and ≥80 cm for women [26,59,60].

WC is a clinically useful marker for abdominal adiposity and cardiometabolic risk. It reflects central fat accumulation and correlates with VAT. Large cohort and meta-analytic data show that WC is independently associated with cardiovascular and all-cause mortality [6,62,63,64,65,66]. In a meta-analysis of over 80,000 individuals from nine cohort studies, WC was significantly associated with cardiovascular mortality, while BMI was not after full adjustment [66]. This suggests that abdominal fat may be more important than overall body mass for cardiometabolic risk assessment. These findings are supported by pooled analyses of more than 650,000 adults, which demonstrated a strong, linear association between WC and mortality across all BMI categories [65].

Much of the clinical value of WC comes from its association with VAT. It does not directly measure VAT, but it is a useful surrogate marker. Evidence shows that decreases in WC are usually accompanied by reductions in VAT [63,65]. Because it is simple, inexpensive, and clinically informative, WC should be routinely assessed in practice. It adds important prognostic information, even in individuals with normal BMI, and improves risk assessment when used together with BMI [8,59].

Despite its usefulness, WC has several limitations. Although it correlates with VAT, the relationship is imperfect. Studies suggest that WC explains only a moderate proportion of the variance in VAT [63,67]. Also, WC does not distinguish between SAT and VAT. Two individuals with the same WC may have different fat distribution and different cardiometabolic risk [62,63]. As discussed earlier, multiple cut-off values exist, and ethnic differences in body composition mean that the same WC may reflect different levels of risk across populations [26,60,62]. Differences in measurement technique can lead to inconsistent results [59]. Another practical disadvantage is that WC measurement can be influenced by postprandial abdominal distension and requires the removal of clothes [68,69,70].

Finally, although most studies suggest that WC is a better marker than BMI for abdominal obesity, metabolic syndrome, CVD, and all-cause mortality, current evidence supports the use of these measures as complementary methods for a more accurate assessment of individual risk [8,59,62].

### 4.2. Waist-to-Hip Ratio

WHR represents the ratio between WC and hip circumference. WC is measured as described above, while hip circumference is measured at the widest point over the buttocks [59]. Both measurements should be performed twice. If the difference is ≤1 cm, the average value is recorded. If the difference is >1 cm, the measurements should be redone. Proposed thresholds differ between populations, although the WHO suggests values of ≥0.90 for men and ≥0.85 for women to define increased metabolic risk [59,71].

WHR provides an indirect estimate of body fat distribution. Higher values suggest greater abdominal fat accumulation, which was associated with increased cardiometabolic risk [8,59,72,73,74]. Meta-analyses have shown that indices of abdominal obesity, especially WHR, can predict cardiovascular events [74]. Studies have also demonstrated that WHR is associated with mortality. In a large 2023 cohort analysis including 387,672 participants of British white ancestry, WHR showed a consistent and linear association with all-cause mortality, independent of BMI [75]. Another meta-analysis reported a significant association between increased WHR and myocardial infarction [76].

When compared with other anthropometric indices across studies, WHR shows mixed results. Some studies suggest that WHR predicts cardiovascular outcomes similarly to or slightly better than BMI [8,72,73,74,75,77]. Other analyses show that WC and BMI have a better correlation than WHR with components of metabolic syndrome [71,78]. Overall, the differences between these indices are relatively small, and their performance may vary according to sex, age, and ethnicity [77,78].

WHR also has important limitations. It should be noted that WHR may remain unchanged when both WC and hip circumferences change in the same direction, which limits its usefulness for monitoring changes over time. In addition, hip circumference reflects not only fat but also muscle mass and skeletal structure, which can influence WHR independently of adiposity [62,79]. WHR is less strongly correlated with total body FM and may be less accurate in identifying components of metabolic syndrome compared to WC or BMI [71,80].

Despite these limitations, WHR provides useful information about fat distribution. In clinical practice, current evidence generally favors other anthropometric indices, particularly WC and WHtR, for risk assessment.

### 4.3. Waist-to-Height Ratio

WHtR is calculated by dividing WC by height, with both measurements in the same units. It is used to assess central adiposity while adjusting for height [71].

Because it adjusts WC for height, WHtR is increasingly used in obesity assessment. One advantage is the use of a single cut-off across different populations [71]. A value of approximately 0.5 was widely proposed as a cut-off to identify increased cardiometabolic risk. This threshold is based on the concept that WC should be less than half of an individual’s height [71,81,82]. A large systematic review reported similar performance of this threshold across different populations, sexes, and age groups. These findings support its use in population screening [81].

This index indirectly reflects abdominal fat accumulation and was linked with cardiometabolic risk factors, including T2DM, hypertension, dyslipidemia, and CVD [83,84,85,86]. Systematic reviews and meta-analyses showed that WHtR performs better than BMI for detecting cardiometabolic abnormalities [81,84,85,86]. In some analyses, this index also performed slightly better than WC, although the differences are modest [81,85]. Overall, WHtR usually performs better than BMI and similarly or slightly better than WC and WHR [81,84,85].

Cohort studies also support its clinical relevance. WHtR was associated with incident hypertension, as shown in the ARIRANG study. Higher values of WHtR significantly increased the risk of developing hypertension over time [83]. In patients with T2DM, the same index has shown a stronger association with cardiovascular and cerebrovascular events compared to BMI, WC, and WHR [86]. Meta-analyses also linked higher WHtR to increased all-cause mortality and cardiovascular mortality [87].

WHtR was also studied in other clinical contexts. A systematic review and meta-analysis from 2023 showed that it has moderate diagnostic accuracy for non-alcoholic fatty liver disease. In this study, significantly higher WHtR values were observed in affected individuals compared to controls [88].

A related index, waist divided by height^0.5^ (WHT.5R), was proposed to further adjust WC for height using allometric scaling. The aim was to reduce the influence of height on risk prediction [6,89]. Some studies suggest that WHT.5R may be a stronger predictor of waist adiposity and combined cardiometabolic risk factors compared to WHtR and other indices [89,90]. However, evidence remains limited, and its clinical utility is less established compared to WHtR [6,89,90].

WHtR also has several limitations. This index accounts for height, but the relationship between height and cardiometabolic risk is complex. Adjusting for height may not always improve risk prediction [6]. Although the 0.5 cut-off is widely supported, optimal thresholds may still vary slightly across populations and clinical contexts [71,81,82].

In clinical practice, WHtR is a simple and low-cost method for assessing obesity. The single cut-off makes it attractive for screening and population-based settings [71,81,84,85].

### 4.4. Neck Circumference

Neck circumference (NC) is an anthropometric measure that reflects upper-body SAT accumulation. NC was investigated as an alternative marker of overweight, obesity, and cardiometabolic risk. Unlike BMI, it provides indirect information on fat distribution. Compared to WC, it is less influenced by postprandial abdominal distension and can be measured quickly without removing clothes [68,69,70,91,92]. These practical advantages make NC attractive for screening in both clinical practice and epidemiological studies [70,91,92,93].

Several studies have reported that NC correlates positively with BMI, WC, and other adiposity-related indices [69,91,92,94,95,96]. Recent data reported associations between NC and body fat percentage, FM index and other body composition parameters related to obesity [94,96]. NC was also associated with multiple cardiometabolic risk factors, including blood pressure, total cholesterol, LDL cholesterol, triglycerides, fasting glucose and insulin resistance [91,93,95]. Associations between NC and VAT and metabolic syndrome were also reported [91,93,96].

Compared to WC, NC had a similar performance in identifying metabolic disorders, which suggests that it may be a reasonable alternative screening tool [96]. However, the performance of its diagnostic accuracy is not uniform across populations [69,70,92,94]. In adolescents and children, NC may be a reliable screening measure, but sex-specific and age-specific cut-offs are usually needed [69,70,92]. In adults, a systematic review and meta-analysis found that NC was accurate for identifying overweight and obesity in most assessments. Performance was generally better in women and individuals with obesity [70].

NC also has important limitations. Cut-off values are not standardized and vary according to age, sex, ethnicity, and study population, although several studies have proposed sex-specific cut-offs (generally around 37–41 cm in men and 32–36 cm in women) [69,70,92,94,97,98,99]. Accuracy seems lower in some groups, particularly in younger children and, in men, where muscle and other non-adipose tissues contribute more to neck size [70,94]. Another disadvantage is that NC does not distinguish VAT from SAT and should not replace more precise body composition or imaging methods [70,93,96].

NC may be useful for rapid obesity and cardiometabolic risk assessment, especially when WC measurement is less practical [70,91,96].

### 4.5. Skinfold Thickness and Mid-Upper Arm Circumference

Skinfold thickness (SFT) is a practical way to estimate body fat percentage. It involves measuring SAT at specific sites, such as the triceps, biceps, subscapular, and supra-iliac regions, with calipers [100]. These measurements are used in prediction equations, such as those developed by Durnin and Womersley and by Jackson and Pollock, to estimate total FM [101,102,103].

SFT has good discriminatory power for identifying overweight and obesity [104,105,106]. SFT was also associated with several cardiometabolic outcomes [102,107,108,109]. Higher skinfold thickness, especially subscapular, was linked to a higher risk of T2DM and hypertension in longitudinal studies [102]. Composite skinfold measures also correlate with cardiometabolic risk factors, including insulin resistance, hyperglycemia, and dyslipidemia [105,109]. This method is inexpensive, portable and non-invasive. It is less affected by daily activities, eating, or changes in hydration than other methods [110]. These qualities make it a good choice for children, athletes, field studies, and clinical situations where more advanced methods are not available [100,103,109].

The accuracy of SFT results depends a lot on the examiner’s experience, the measurement protocol, and the prediction equations used [101,103,108]. Using different types of calipers can cause systematic errors and make it harder to compare results between studies [111]. Another drawback is that it focuses mainly on SAT and does not capture VAT [8,103,109].

Mid-upper arm circumference (MUAC) is another anthropometric measurement used in obesity assessment. It was studied as a marker of overweight, obesity, central adiposity, and insulin resistance in both adults and children [112,113,114,115]. It is simple, inexpensive and requires minimal equipment. MUAC is used in clinical practice when accurate height measurement is not possible, and BMI cannot be calculated, or accurate weight is affected by fluid retention in the body. Unlike BMI and SFT, MUAC does not require additional calculations. The proposed cut-off for predicting central obesity is a MUAC ≥ 30.9 cm for males and ≥30.0 cm for females. However, optimal cut-off values for MUAC can vary by age, sex and population characteristics [112].

Anthropometric indices provide additional information beyond BMI, especially regarding fat distribution and cardiometabolic risk. WC and WHtR are among the most practical methods for routine clinical use, while WHR, NC, SFT, and MUAC may provide complementary information in selected populations or clinical settings. However, these methods estimate adiposity indirectly and cannot distinguish VAT from SAT. Because of these limitations, body composition techniques and imaging methods are often needed for a more detailed assessment of obesity. The next section will focus on these techniques.

## 5. Body Composition Methods Beyond Anthropometry

**Figure 1:**
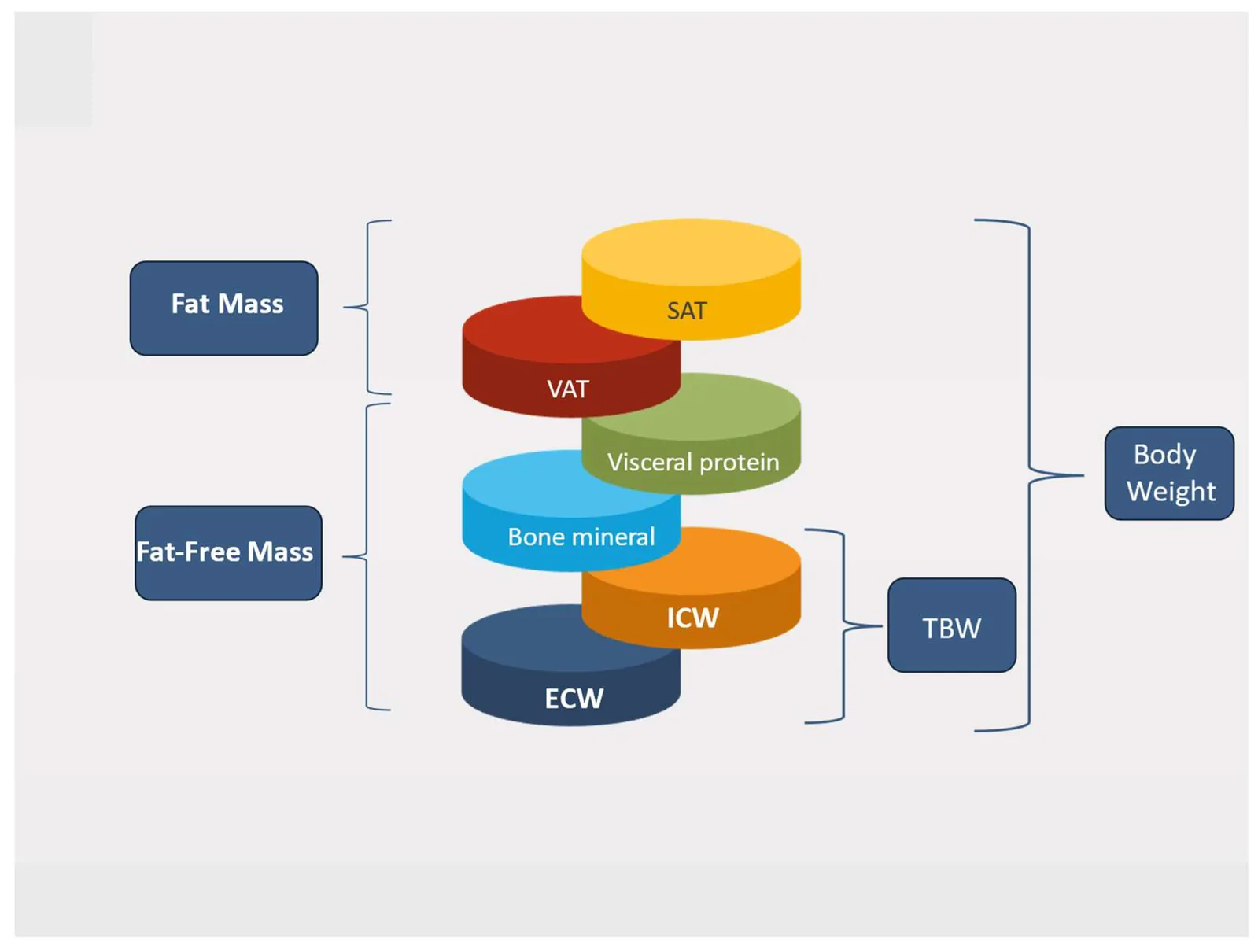
Simplified representation of body composition compartments relevant for obesity assessment. Body weight is divided into Fat Mass and Fat-Free Mass. Fat mass includes subcutaneous adipose tissue (SAT) and visceral adipose tissue (VAT). Fat-free mass consists of total body water (TBW), further divided into intracellular water (ICW) and extracellular water (ECW) compartments, visceral protein and bone mineral.

Anthropometric methods are useful for the initial assessment of obesity, but they estimate adiposity indirectly. Body composition techniques provide additional information by separating FM and FFM. Some methods also estimate regional fat distribution and can differentiate SAT from VAT. As shown in Figure 1, body weight consists of multiple compartments, including FM and FFM [116,117,118,119]. For example, as discussed in the BMI section, changes in body weight do not necessarily represent changes in FM, as weight loss may also result from reductions in FFM. Body composition methods help distinguish between these components and provide a more detailed assessment of obesity compared with anthropometric indices alone.

### 5.1. Bioelectrical Impedance Analysis

A popular non-invasive method to evaluate body composition is BIA. The method developed from early observations that biological tissues have measurable electrical properties. In early studies, impedance was utilized as an index for total body water (TBW) [117]. Later, the introduction of the four-surface electrode technique made the method practical for clinical use. The fundamental principles of BIA were defined in 1970s and in the following decades single-frequency and then multifrequency devices became widely available [117,118,120].

Electrical current passes more easily through tissues rich in water and electrolytes, such as lean tissue, and less easily through adipose tissue [116,117,118]. Electrodes are placed in contact with the body, often in a tetrapolar arrangement on the hand and leg. The device measures the opposition of the body to the current, called impedance [118]. Because the human body is not a perfect cylinder, BIA does not directly measure body compartments. For this reason, impedance values are used in prediction equations, usually together with height, weight, age, and sex, to estimate FFM, FM, and TBW [117,118].

Several types of BIA are currently used. Single-frequency BIA operates at 50 kHz and is mainly used to estimate FFM and TBW. The inconvenience is that TBW is determined by the sum of ICW and ECW and is less reliable in altered hydration status [116,117,119]. Multifrequency BIA uses several frequencies and provides more information about body water distribution, including TBW, ECW and ICW compartments, even in fluid retention or malnutrition status [116]. In older adults, its ability to detect changes in ECW and ICW is weak [117,119,120]. Bioelectrical impedance spectroscopy uses mathematical modeling across a range of frequencies to estimate body fluid compartments [117,120]. Although the trunk is about 50% of total body mass, it contributes only 10% of total body impedance. Segmental BIA was developed to overcome this limitation and provide regional estimates [117,119].

BIA is non-invasive, rapid, and relatively easy to use, which explains its widespread use in clinical practice and large studies [121]. The method is safe and does not expose individuals to ionizing radiation. It can be used multiple times in both clinical and research contexts [8]. BIA is also widely accessible and relatively inexpensive compared with reference techniques such as Dual-Energy X-Ray Absorptiometry (DXA) or Computed Tomography (CT) [8]. It is not dependent on operator expertise, and portable devices allow bedside and field assessments [118]. As mentioned above, the technique can estimate multiple body composition compartments, including FM and FFM, providing more information than anthropometric indices and facilitating more detailed characterization of obesity [121,122].

In clinical practice, BIA can detect conditions such as sarcopenia and sarcopenic obesity that may be missed when using BMI [122]. Parameters measured by BIA, such as body fat percentage and FM have been shown to correlate with morbidity, mortality outcomes and cardiovascular risk [121]. Modern multifrequency BIA devices have very high reliability (ICC ≥ 0.99) and minimal day-to-day variation [116,120,123,124]. These results support the use for longitudinal monitoring of body composition changes. Overall, BIA provides reproducible results with acceptable agreement with reference methods for routine clinical assessment [124,125]. Compared with other convenient techniques in clinical practice, BIA shows better performance for body composition estimation and is more sensitive to changes over time, an aspect where anthropometric methods often lack precision [124,126]. Furthermore, it can generate reasonably accurate estimates of visceral fat area and may represent a convenient alternative to imaging techniques such as CT [127,128].

Despite its practical advantages, BIA remains an indirect method of body composition assessment. It is based on predictive equations derived from electrical impedance and population-based assumptions, so it does not measure FM and FFM directly [8,124,129]. Its accuracy is dependent on the selected equation, and equations developed in one population may not perform well in others [118,124,129]. Hydration is a major source of error because impedance is strongly influenced by body water and electrolyte distribution. Therefore, the method is less reliable in conditions such as edema, ascites, dehydration, pregnancy, kidney or liver disease, heart failure, and other states with altered fluid balance [8,116,129,130]. Results are also affected by factors such as food intake, recent physical activity, temperature of ambient and skin, and electrode placement, which is why standardized measurement conditions are important to obtain reliable measurements [116,124,129]. Multiple studies reported systematic differences between BIA and DXA, most often overestimating FFM and underestimating body fat percentage, which means that the two methods should not be considered interchangeable [8,120,123,131]. Recent work by Potter et al. suggests that applying +3% correction to body fat percentage may improve agreement with DXA [120,123].

Despite these limitations, BIA remains widely used because it is rapid, non-invasive, relatively inexpensive and reproducible. Although less accurate than reference imaging methods, it provides useful information about body composition in both clinical practice and research settings.

### 5.2. Dual-Energy X-Ray Absorptiometry

Since its introduction in the late 1980s, DXA has become one of the most commonly used techniques for evaluating bone mineral density and body composition [132,133]. DXA uses two X-ray beams with different energy levels to differentiate body tissues. Differences in X-ray attenuation are used to estimate three compartments: FM, lean soft tissue (LST), and bone mineral content [118,134]. These compartments are estimated from scan-derived mathematical models rather than measured directly [118,134].

DXA allows both whole-body and regional analysis, including limbs, trunk, and predefined regions such as android and gynoid areas [132,134]. Indices such as appendicular lean mass (ALM/height^2^) or fat distribution ratios can be derived from these measurements and used in sarcopenia and cardiometabolic risk assessment [135,136].

DXA has good precision and reproducibility in body composition assessment. Reported coefficients of variation are approximately 0.8–2.7% for FM and around 1% for LST in whole-body measurements [118,134]. Because of this precision, DXA is frequently used in longitudinal monitoring. Scan time is relatively fast. Whole-body scans typically take around 5 min, and even 1 min for regional scans [134]. Radiation exposure is low, with less than 10 μSv delivered in a whole-body scan. This exposure is comparable to approximately one day of natural background radiation. It also represents only about 1–10% of the exposure received from a chest X-ray. Radiation exposure is considerably lower than CT [134,136].

DXA shows better accuracy than anthropometry and BIA for estimating FM and LST [8,134,137]. It has good correlation with CT and MRI measurements, but it lacks the same level of anatomical detail [133,135]. Because of this, DXA is often used as a reference technique in body composition research [119].

At the same time, DXA also has several limitations. Similar to BIA, DXA does not measure body compartments directly. It is based on assumptions such as constant hydration of FFM and uniform soft tissue composition that can reduce accuracy in certain physiological or clinical conditions [118,133,134]. Unlike CT or MRI, it cannot directly separate different fat depots. VAT is estimated indirectly, usually from the difference between total FM and SAT, which may reduce accuracy [8,63]. Some systematic differences have been described in the literature. DXA tends to underestimate FM in lean individuals and overestimate it in those with higher adiposity [118,134]. LST may also be overestimated, partly because the method cannot distinguish muscle from other components of lean tissue [118,134,137]. At an individual level, variability can be considerable, with reported differences in body fat percentage ranging from approximately −2.6% to +7.3% compared with four-compartment reference models [134].

Measurement accuracy is affected by practical factors. Greater body thickness, especially above 25 cm, can alter X-ray attenuation and affect measurement accuracy in individuals with obesity [134]. Results may also vary with positioning, scan protocol, and device-specific factors. Differences between scanners or software versions can reach up to 7% for FM and 4% for FFM [118,119,134].

DXA also has practical limitations. The method is not portable, requires trained personnel, and is not always available for body composition analysis outside specialized centers [119,134]. Table weight and size limits can restrict its use in severe obesity, but newer systems have improved capacity [134]. Even if the radiation dose is low, its use is generally avoided during pregnancy [135].

In practice, DXA offers more detailed body composition analysis than anthropometry or BIA, but less detailed than CT or MRI. Its relatively high accuracy, short examination time, and low radiation exposure explain why DXA remains widely used in both research and clinical practice.

## 6. Imaging Methods for Adiposity Assessment

### 6.1. Computed Tomography

CT provides highly accurate in vivo assessment of body composition. Cross-sectional CT images allow direct quantification of adipose tissue and skeletal muscle [118,138]. In practice, CT-based body composition analysis (CTBC) is often derived from scans already performed for diagnosis or follow-up [139]. This allows opportunistic assessment without additional imaging. Most analyses are performed at the level of the third lumbar vertebra (L3), because measurements at this level correlate well with whole-body fat and lean mass (r ≈ 0.86–0.94) [140].

In the context of obesity, CT allows direct measurement of VAT and clear separation from SAT. This is not possible with anthropometric measures such as BMI or WC, or with methods like BIA, which only provide indirect estimates of visceral fat area [63,118,134]. This is clinically relevant, as VAT shows a stronger relationship with cardiometabolic risk than total adiposity [10,13]. CT was also used to develop ethnicity- and sex-specific prediction models for VAT and SAT in large multi-ethnic cohorts, supporting the role of VAT and SAT assessment in cardiometabolic risk evaluation [141]. Increased VAT measured by CT was linked to metabolic disease, atherosclerosis, and worse clinical outcomes, independently of BMI. In the Health ABC cohort, direct CT assessment of muscle area showed a stronger association with all-cause mortality than DXA-derived lean mass [142,143].

CT is associated with a significant radiation burden. Abdominal examinations are typically associated with effective doses in the range of approximately 5–10 mSv. This is roughly equivalent to several hundred chest radiographs [134,144]. With its wide use, CT now accounts for a large share of medical radiation exposure [144]. A small increase in lifetime cancer risk cannot be excluded, and epidemiological data have linked CT exposure to higher rates of certain malignancies [145,146]. At the population level, it was estimated that up to 1.5–2.0% of cancers in the United States may be attributable to CT-related radiation [147]. These considerations limit repeated use and make CT difficult to justify when performed solely for body composition assessment.

### 6.2. Magnetic Resonance Imaging

MRI considerably improved the evaluation of body composition. Its high resolution allows detailed assessment of adipose tissue and skeletal muscle, which is why it is often considered a reference imaging technique [134]. MRI uses proton density and T1/T2 relaxation to produce high-contrast images without radiation. Multislice imaging is a gold standard, but, in practice, single-slice protocols are often preferred because they are simpler, more accessible and cheaper [8].

In obesity assessment, MRI can distinguish between fat depots, including SAT, VAT, intermuscular, and ectopic compartments [118]. These tissue compartments and muscle alterations cannot be evaluated with BMI. Individuals with similar BMI may have different metabolic risk and MRI allows a more detailed characterization of obesity, based on tissue distribution rather than body size alone [134,148]. At the same time, MRI quantifies muscle volume and quality, which is particularly relevant in the context of sarcopenic obesity [118].

More advanced MRI techniques provide additional metabolic information. Dixon-based sequences separate fat and water signals and generate voxel-level fat fraction maps [149]. MR spectroscopy can further quantify lipid content in the liver and muscle, providing insight into metabolic alterations such as insulin resistance [134,149].

Compared with DXA, MRI offers true three-dimensional assessment of adipose tissue and muscle distribution. In the 2026 UK Biobank analysis, DXA estimates fat-related measures reasonably well, but overestimates LST compared with MRI [133]. Longitudinal MRI detects a 4–5% decline in muscle mass that DXA may miss [133]. This makes MRI particularly useful when obesity is accompanied by loss of muscle mass or suspected sarcopenic obesity [133,149].

The major advantage of MRI compared with CT is the absence of ionizing radiation [71,134]. This makes it suitable for repeated measurements and longitudinal studies [134]. Despite this difference, MRI and CT provide comparable body composition measurements. In a same-day prospective study, CT and MRI measurements of paraspinal muscle area were strongly correlated (r = 0.93) and indicators of muscle fat content were closely related between methods, with CT attenuation values corresponding well to MRI-derived fat fraction (r = −0.90) [150]. Zaffina et al. also reported strong correlations between CT and MRI for skeletal muscle index, SAT, and VAT, with the highest agreement observed for VAT (r^2^ = 0.93–0.94) [151]. These studies show that CT and MRI provide similar estimates of body composition, although MRI remains preferable for repeated obesity phenotyping because it avoids radiation [134,149,150,151].

Even if MRI is considered a reference method, it is not used routinely for obesity assessment in most clinical settings. It is expensive and not widely available. It also requires trained personnel and more complex post-processing [8,134]. Semi-automated and AI-based tools are helping, but they are not always standardized [149].

There are also practical limits, especially in severe obesity. Scanner tables have weight limits, and bore diameter can be restrictive. In some cases, body size exceeds the field of view [8,134]. MRI scans also take longer, usually from tens of minutes to over an hour [152]. Other factors matter too: claustrophobia, difficulty staying still, or contraindications such as ferromagnetic implants [134].

In current practice, MRI is used mainly for research purposes and detailed obesity phenotyping rather than a first-line method for routine clinical screening [63,71,134].

### 6.3. Ultrasound

Ultrasound is increasingly investigated as a tool for body composition assessment. It is based on the reflection of sound waves at tissue interfaces, so it allows differentiation between adipose tissue, muscle, and bone [134]. It can also be used for direct measurement of SAT, muscle thickness, and intra-abdominal fat thickness [118].

Both A-mode and B-mode ultrasound can be used. A-mode is more basic; it shows tissue interfaces as spikes on a graph rather than an image. B-mode gives an actual image, a grey-scale one, and is the one used more often in body composition [118].

When standardized protocols are used, ultrasound shows good validity and reproducibility for SAT assessment [118]. In the work by Wagner, strong correlations were reported between SAT measured by ultrasound and body fat percentage, with r values approaching 0.90–0.95 when multiple sites were used [153]. Even a single abdominal measurement can still give useful information. Data from Torgutalp et al. indicate a reasonable level of agreement with DXA-derived total FM (concordance correlation coefficients of 0.84 in men and 0.76 in women), although this seems to vary depending on the population studied [154]. For VAT, ultrasound usually relies on measuring intra-abdominal fat thickness, using distances between landmarks like the aorta, the lumbar spine or the abdominal wall [8,155].

The correlations with VAT measured by CT are, in most cases, moderate. Values are often somewhere around r ≈ 0.65–0.74, with some reports closer to r = 0.67 [8,153,156].

Still, the findings are not very consistent. Part of the issue comes from how the measurements are taken, and how these anatomical limits are defined in different studies [134,155]. In some papers, variability is actually quite high. Coefficients of variation can reach around 60%, which makes the method less reliable, especially at the individual level rather than in groups [8,153].

Ultrasound allows quick, site-specific measurements, usually taking only seconds or a couple of minutes per region, and without radiation [134]. It is portable and can be applied in obese subjects or in areas not accessible for skinfold calipers [118]. Compared to other methods, ultrasound provides regional fat distribution data not accessible by anthropometry and avoids the radiation exposure associated with CT or DXA [134].

Overall, ultrasound performs well for SAT assessment, while the accuracy for VAT estimation is more moderate. Its clinical utility is still limited, mainly because it is operator-dependent and standardization is not well established [134].

### 6.4. Three-Dimensional Optical Imaging

Three-dimensional optical (3DO) imaging is a relatively recent digital anthropometric technique [157]. In recent years, the technology has improved significantly. Devices have become faster, easier to use, and more accessible in practice [157,158]. The method does not measure internal tissues directly, but it captures the external surface using optical systems and builds a 3D model [157]. From this, the software can derive circumferences, lengths, volumes, and multiple body shape parameters [157,159].

One advantage is the amount of information it provides compared to simple anthropometric methods [157]. In the Shape Up! dataset, 3DO estimates were very close to DXA for both FFM and FM, with concordance values around 0.97 and 0.95 [160]. Similar results were reported using statistical shape modeling, where FM estimates showed good agreement with DXA, with R^2^ values around 0.88 in men and 0.93 in women [159]. There is also increasing interest in using 3DO for follow-up, not just single assessments [158]. In longitudinal intervention data, changes estimated by 3DO correlated well with DXA. This makes it useful for repeated monitoring, because scans are quick and radiation-free [158,159]. Cost is also a factor. 3DO is generally considered more accessible and less expensive than methods like DXA or CT [157]. This partly explains its growing use.

Estimating composition from body shape, not from direct tissue measurement, is the main limitation of 3DO [157]. Because of this, results depend on several factors. The scanner itself, the prediction model, subject position, and clothing can all influence accuracy. The population used to build the model also affects the results [157,159]. VAT can be estimated, but performance is weaker than for FM. Reported R^2^ values are around 0.67 in men and 0.75 in women when compared with DXA [159]. There are also some practical issues. In individuals with obesity, body contours are less clearly defined. This can make landmark detection more difficult and may affect measurements [157].

3DO is probably better seen as an advanced form of anthropometry. It gives more detail, and it is useful for tracking changes over time. But it does not replace methods like DXA, CT, or MRI [157,159].

## 7. Clinical Integration of Obesity Assessment Methods

Considering its high prevalence, obesity assessment should not rely on a single parameter in clinical practice. BMI is widely used for initial screening because of its simplicity, but it is far from sufficient. It provides no information on body composition or fat distribution [15,42]. For this reason, complementary methods beyond BMI are needed to confirm excess adiposity, assess its distribution, and better define individual risk profiles. No single method is perfect, without limitations, so in clinical practice it is important to consider the context and to identify when each method is most appropriate.

The performance of these approaches cannot always be compared directly. Sensitivity and specificity depend on the population, cut-off values, definition of obesity or excess adiposity, and the reference method used. For body composition and imaging techniques, validation is also frequently reported through measures of agreement, correlation, or precision.

A comparative overview of these methods, including their performance data, main characteristics and clinical applications, is presented in Table 1.

In practice, obesity assessment methods are usually selected according to their clinical role. Anthropometric indices are primarily used for screening and initial risk assessment [59,77,78]. These methods are inexpensive, fast, easy to apply, and suitable for routine clinical settings. Among them, WC and WHtR show some of the strongest associations with cardiometabolic risk, mainly because of their relationship with abdominal adiposity [63,65,81,85].

Body composition techniques such as BIA and DXA allow a more detailed assessment of obesity, going beyond BMI or anthropometry. They estimate FM and FFM, and can also provide information on how these are distributed across the body, for example between the trunk and the limbs. These methods become particularly useful when a more precise evaluation is needed, especially in individuals where BMI does not match the metabolic profile, or in the evaluation of phenotypes such as sarcopenic obesity. In practice, BIA is more common, easier to access and better suited for routine use and follow-up, while DXA provides more accurate and detailed regional data but is more often used in specialized settings or research [8,121,134].

Advanced imaging techniques, such as CT and MRI, offer the most detailed assessment of body composition in obesity and are often considered reference methods. They allow direct measurement of VAT and provide a precise evaluation of muscle mass and ectopic fat [138,140]. In practice, their routine use is limited by cost and availability, and in the case of CT, by exposure to ionizing radiation [144]. For this reason, CT is usually used opportunistically, based on scans performed for other clinical purposes, while MRI is generally reserved for research settings or for selected cases requiring a more detailed evaluation [63,71,134].

Adjunctive techniques, such as ultrasound and 3DO, are additional options for body composition assessment in obesity. They are cheaper and easier to implement than DXA, CT, or MRI and are comparable to BIA in terms of feasibility in routine settings. Their agreement with these methods is acceptable when estimating FM or SAT, but their performance is more limited for VAT [157,158]. This limits their ability to fully characterize obesity-related metabolic risk [8,10,11]. In practice, their role is additional when access to more advanced imaging methods is limited or when repeated assessments are required.

**Figure 2:**
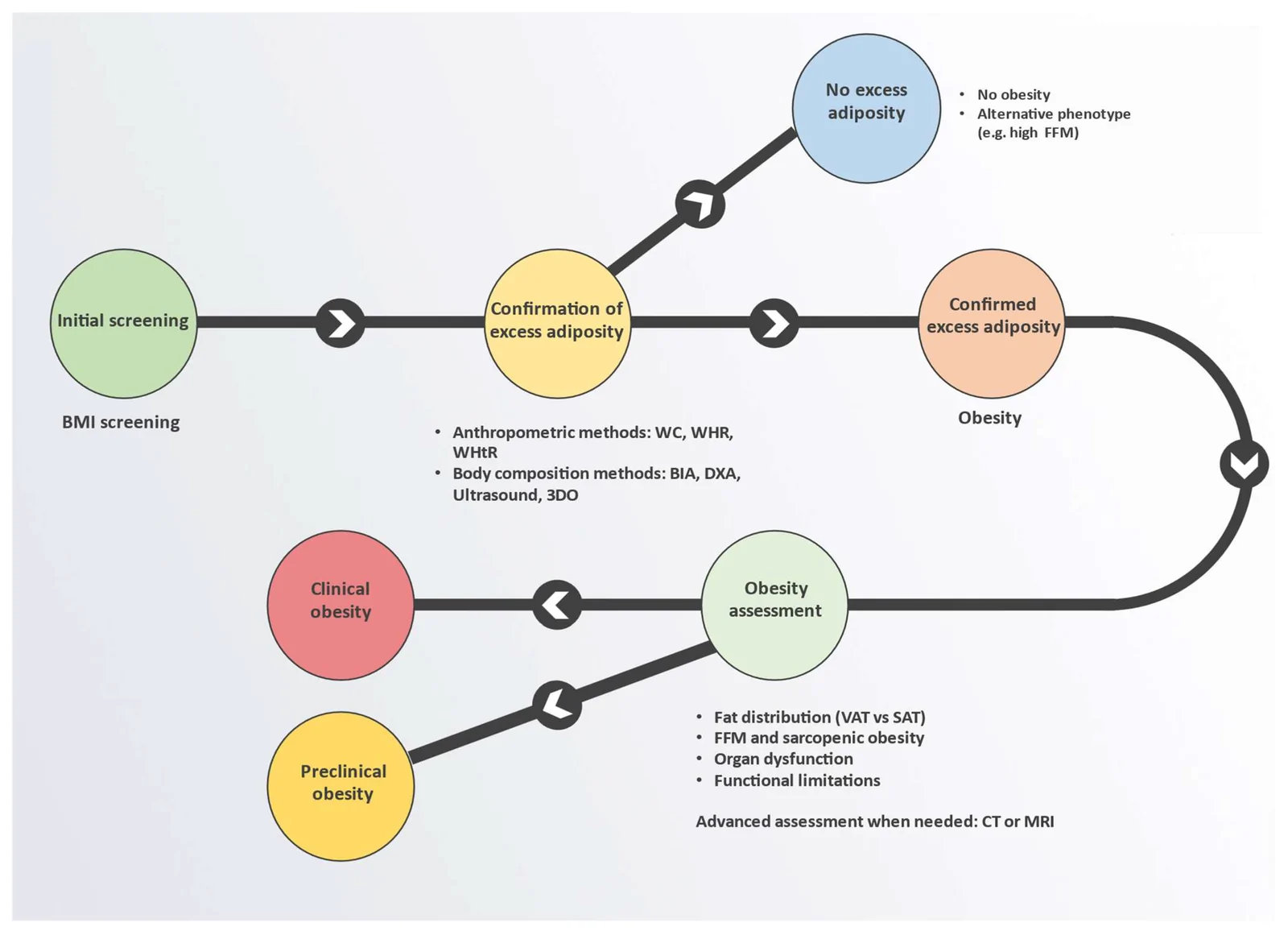
Simplified framework for clinical obesity assessment beyond BMI. Abbreviations: BMI, body mass index; WC, waist circumference; WHR, waist-to-hip ratio; WHtR, waist-to-height ratio; BIA, bioelectrical impedance analysis; DXA, dual-energy X-ray absorptiometry; 3DO, three-dimensional optical imaging; FFM, fat-free mass; SAT, subcutaneous adipose tissue; VAT, visceral adipose tissue; CT, computed tomography; MRI, magnetic resonance imaging.

Discordance between BMI and measures of central adiposity deserves particular attention. For example, a patient with a BMI of 31 kg/m^2^ but a normal WC may benefit from body composition assessment with BIA or DXA. This can help determine if the high BMI reflects excess adiposity or a greater amount of FFM [15,18]. The opposite situation is also relevant. A patient with a BMI below 30 kg/m^2^ but an increased WC or WHtR may still have excess central adiposity and increased cardiometabolic risk [17,18]. In this case, another anthropometric measure or body composition assessment can provide additional confirmation and better characterize adiposity [15,18]. More advanced imaging is usually unnecessary at this stage and should be reserved for specific clinical questions, such as detailed assessment of VAT, ectopic fat, or muscle composition [18,19]. Method selection often reflects the trade-off between accessibility and diagnostic detail in obesity assessment. Based on these considerations, a simplified framework illustrating their integration in clinical practice is shown in Figure 2.

The framework follows the direction suggested by the recent Commission on Clinical Obesity published in 2025 [15,16]. These recommendations emphasize that BMI should be used primarily as a screening tool and not as a sufficient diagnostic criterion for obesity. Confirmation of obesity requires additional evidence of excess adiposity, obtained through other anthropometric indices or through body composition assessment methods. The Commission also proposed separating preclinical obesity from clinical obesity. The diagnosis of clinical obesity is not based only on adiposity, but also on its impact on organ function and daily functioning [15,16].

**Figure 3:**
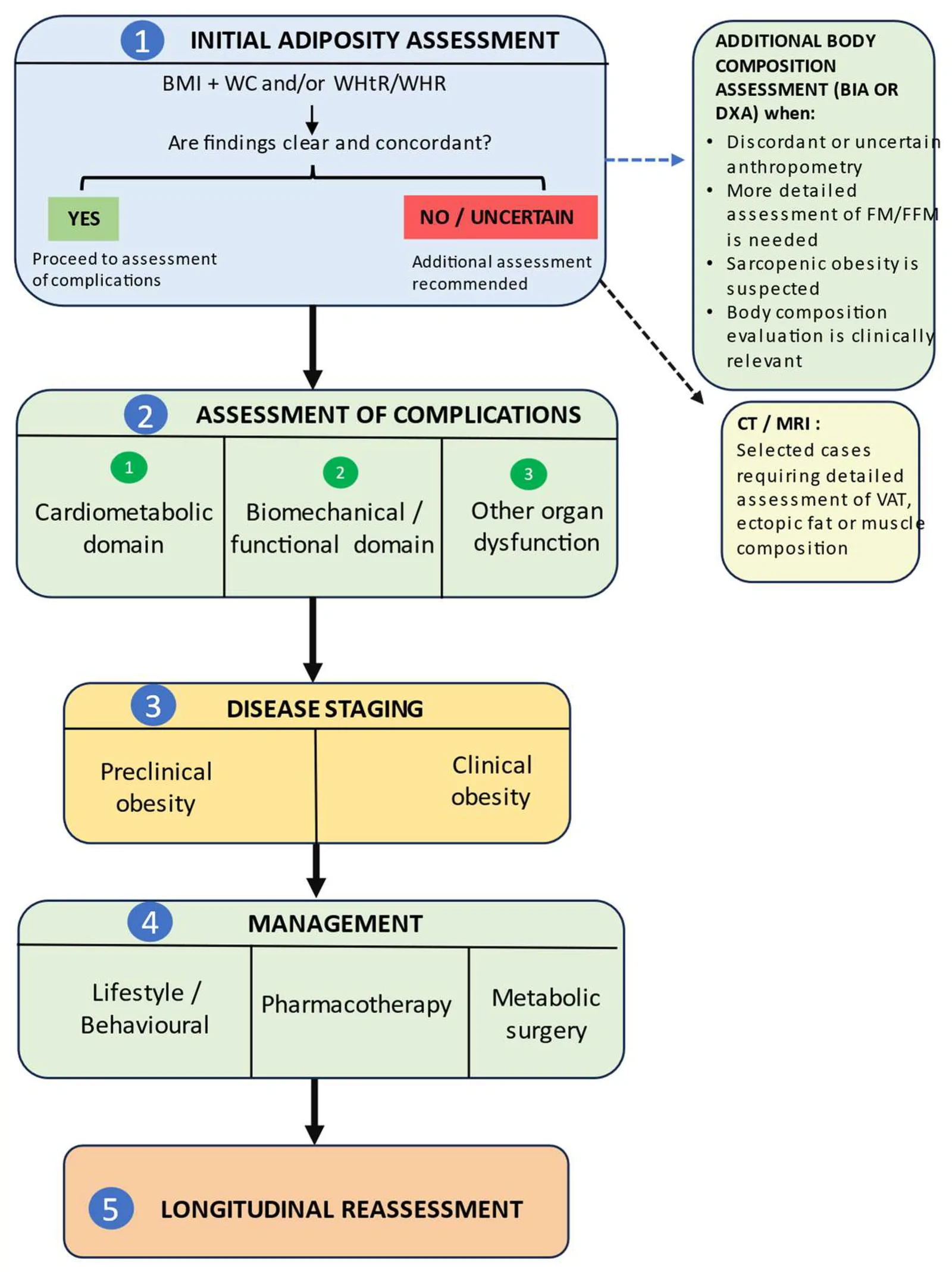
Practical clinical pathway integrating adiposity assessment, obesity complications, disease staging, management, and longitudinal reassessment. Abbreviations: BMI, body mass index; WC, waist circumference; WHR, waist-to-hip ratio; WHtR, waist-to-height ratio; BIA, bioelectrical impedance analysis; DXA, dual-energy X-ray absorptiometry; FM, fat mass; FFM, fat-free mass; VAT, visceral adipose tissue; CT, computed tomography; MRI, magnetic resonance imaging.

Once excess adiposity is confirmed, the next step is to assess its clinical impact. Organ dysfunction may involve several systems. Examples include hypertension, heart failure or recurrent atrial fibrillation, obstructive sleep apnea or obesity-related hypoventilation, metabolic abnormalities, metabolic dysfunction-associated steatotic liver disease with fibrosis, and renal dysfunction. Reproductive abnormalities, such as anovulation, polycystic ovary syndrome or male hypogonadism, may also be present. Musculoskeletal involvement is another relevant manifestation, particularly when severe knee or hip pain limits movement. Functional impairment should also be considered. This includes reduced mobility or difficulty with basic activities of daily living. When sarcopenic obesity is suspected, muscle strength and physical performance should also be assessed. These findings help distinguish preclinical obesity, where organ function is preserved, from clinical obesity, where excess adiposity is associated with organ dysfunction or relevant functional limitation [15,17,18]. Based on these considerations, a practical pathway integrating adiposity assessment, obesity complications, disease staging, management, and longitudinal reassessment is proposed in Figure 3.

Body composition is also relevant when treatment response is evaluated. Recent evidence shows that anti-obesity pharmacotherapy can reduce both FM and lean mass, although the magnitude differs between drugs [170]. This may be particularly relevant in older adults and in patients at risk of frailty or sarcopenia. These findings support looking beyond BMI alone during follow-up [170]. When appropriate, body composition and functional assessment should be included in the evaluation of treatment response.

From this perspective, obesity assessment methods reviewed in this paper can offer more than a simple estimation of body fat or confirmation of excess adiposity. They can help characterize obesity phenotypes more precisely, including sarcopenic obesity, differences in VAT and SAT distribution, and variations in cardiometabolic risk between individuals with similar BMI. Combining anthropometric measures with body composition and imaging techniques may improve obesity assessment beyond BMI. The best combination of methods depends on the setting and the purpose of assessment. In clinical practice, additional methods can be used when more detail is needed, especially when anthropometric findings are unclear or when body composition may influence clinical decisions. In population studies, simple and low-cost methods are usually more practical because they can be applied to large numbers of people. Cost differences between methods can be substantial. Recent estimates place the price of clinical BIA devices between approximately €3000 and €20,000, depending on the system [171]. A whole-body DXA device may cost around $60,000, with an estimated cost of $100–200 per scan [172]. In a 2026 analysis, adding targeted BIA to BMI screening increased the modeled screening cost by 78.4% and reduced screening capacity by 24.3% [173]. These costs can vary between devices, countries, and healthcare systems. This is relevant when assessment methods are considered for routine use or large-scale screening.

Future studies should compare combinations of methods and not only individual techniques. They should also assess whether adding more complex evaluations improves risk prediction, clinical decisions, or patient outcomes. Better standardization of measurement protocols is still needed. Further validation in different age groups, sexes, ethnic populations, and obesity phenotypes would also be useful.

## 8. Conclusions

Obesity assessment in clinical practice requires moving beyond BMI. While useful for screening, BMI misses fat distribution, body composition and the full metabolic picture. Recent guidelines have recognized these limitations. They recommend that excess adiposity should be confirmed using additional anthropometric or body composition measures before establishing the diagnosis of obesity.

The methods discussed in this review are not interchangeable, but rather complementary. Anthropometric indices such as WC, WHtR, and WHR improve the evaluation of abdominal adiposity and cardiometabolic risk. Body composition techniques such as BIA and DXA add information on FM, FFM, and regional fat distribution, including phenotypes such as sarcopenic obesity that may remain undetected when relying only on BMI. CT and MRI offer the highest precision for VAT quantification, though cost and availability still limit their routine use.

In practice, the choice of method depends on the clinical setting and the purpose of evaluation. The traditional BMI-centered approach is no longer sufficient on its own. In line with the growing international consensus, combining anthropometric, body composition, and imaging methods may improve the assessment of adiposity and obesity-related risk.

## Figures and Tables

**Table 1:** Comparative overview of clinical methods for obesity assessment.

Index/Technique	Main Information Provided	Performance	Practical Advantages	Main Limitations	Clinical Use
**Anthropometric indices and measurements**					
BMI	Weight status based on weight and height [5,24]	Se 74.6%; Sp 82.2% vs. DXA [161]	Simple; low-cost; widely used; standardized [5,24,27]	Does not distinguish FM from FFM; does not reflect fat distribution; may misclassify individuals [7,42]	First-line screening; epidemiological studies [24,27]
WC	Indirect estimate of abdominal adiposity [59,62]	Se 88.5%; Sp 66.7% vs. DXA [161]	Simple; low-cost; useful surrogate of abdominal fat; associated with CVD and all-cause mortality [62,65]	Does not distinguish VAT from SAT; affected by technique, abdominal distension and population-specific cut-offs [59,62]	Routine assessment of abdominal obesity and cardiometabolic risk [59,62]
WHR	Indirect estimate of body fat distribution (abdominal vs. gluteofemoral adiposity) [59,71]	Se 90.8%; Sp 42.1% vs. DXA [161]	Reflects fat distribution; associated with cardiometabolic risk, CVD, and mortality [74,75,76]	Limited for monitoring changes; less strongly correlated with total body FM; variable performance across populations [62,71]	Additional assessment of fat distribution [59,74,75]
WHtR	Indirect estimate of central adiposity adjusted for height [74,81]	Se 99.5%; Sp 33% vs. DXA [161]	Simple; low-cost; single cut-off (0.5); consistent performance across populations; stronger association with cardiometabolic risk than BMI and comparable or slightly better than WC [81,84,85]	Optimal thresholds may still vary slightly; height adjustment does not always improve prediction [74,82,83]	Screening and population-based assessment of cardiometabolic risk [81,84,85]
NC	Upper-body SAT; indirect marker of adiposity and fat distribution [91,94]	Se 83–86%; Sp 71–74% vs. DXA [97]	Simple; low-cost; no need for extensive clothing removal; associated with cardiometabolic risk factors [70,91,93]	Lack of standardized cut-offs; variable performance across populations; influenced by muscle and other non-adipose tissues; does not distinguish VAT from SAT [70,94]	Adjunctive screening tool; alternative when WC is less practical [70,91,96]
Skinfold thickness	SAT and estimated body fat percentage [100,103]	Se 57–78%; Sp 69–93% [106,162]	Affordable; portable; less affected by hydration and short-term factors; useful in field settings and large studies [100,103,110]	Operator-dependent; influenced by measurement protocol and prediction equations; does not assess VAT [101,103,111]	Field and epidemiological assessment; estimation of body fat, especially when other methods are not available [100,103]
MUAC	Arm circumference related to adiposity [112,115]	Se 72.8%; Sp 94.4% [163]	Simple; low-cost; does not require weight or height; no calculations needed [112,113]	Cut-offs vary by age, sex, and population; limited information on fat distribution [112,113,114]	Alternative assessment when BMI cannot be calculated or weight/height measurements are unreliable [112,113,114]
**Body composition techniques**					
BIA	FM, FFM, total body water and fluid compartments; indirect estimates of VAT [117,118,120]	Se 72.3%; Sp 94.4% vs. DXA [164]	No radiation; accessible; portable; relatively inexpensive; rapid and reproducible; suitable for repeated measurements and follow-up [118,120,124]	Indirect method based on predictive equations; affected by hydration status, clinical conditions, food intake, physical activity, temperature, and measurement conditions [118,120,124,130]	Routine body composition assessment and longitudinal monitoring; evaluation of sarcopenia and sarcopenic obesity [121,122,130]
DXA	FM, lean soft tissue, bone mineral content; whole-body and regional distribution [118,134,136]	High precision (CV 0.7–3.4% for FM; 0.4–2.2% for LST); SEE 2–3% vs. 4C model [165]	More accurate than BIA; reproducible; short scan time; low radiation dose [118,134]	Indirect method based on assumptions; cannot directly separate fat depots (VAT estimated indirectly); not portable and limited availability [118,134]	Detailed body composition assessment in clinical and research settings; evaluation of regional fat distribution and sarcopenic obesity [119,134,135]
**Imaging and digital techniques**					
CT	Direct quantification of VAT, SAT, skeletal muscle [118,138,140]	Reference method; excellent reproducibility (ICC 0.98–1.00) [165,166]	Highly accurate; direct measurement of VAT with clear separation from SAT; strong association with cardiometabolic outcomes; can be used opportunistically from existing scans [63,138,140]	Expensive; significant radiation exposure; not suitable for repeated use; not justified solely for body composition assessment [134,144]	Opportunistic body composition analysis from clinically indicated scans [139,143]
MRI	Detailed assessment of adipose tissue compartments (SAT, VAT, ectopic fat), muscle volume and quality [118,134]	Excellent repeatability and reproducibility (ICC 0.90–1.00) [167]	High-resolution; no ionizing radiation; detailed phenotyping [134,149]	Expensive; limited availability; longer scan time; practical limits in severe obesity [8,134,149]	Complex cases; detailed obesity phenotyping [63,134]
Ultrasound	SAT thickness, muscle thickness, indirect estimation of intra-abdominal fat [118,134,155]	Se 69.2%; Sp 82.8% vs. CT [168]	Portable; quick, no radiation; lower cost than advanced imaging [118,153]	Operator-dependent; limited standardization; moderate and inconsistent accuracy for VAT; [134,155]	Complementary method for regional assessment, especially when other imaging techniques are not available [134,155]
3DO	Estimates of FM, FFM; external body dimensions [157,159]	FFM/FM: CCC 0.97/0.95 vs. DXA [160,169]	No radiation; quick and reproducible; useful for monitoring; relatively accessible [157,160]	Indirect method based on body shape; weaker estimation of VAT; affected by body morphology and landmark detection [157,159]	Advanced anthropometry; large-scale screening and longitudinal monitoring [157,158]

Abbreviations: BMI, body mass index; WC, waist circumference; WHR, waist-to-hip ratio; WHtR, waist-to-height ratio; NC, neck circumference; MUAC, mid-upper arm circumference; BIA, bioelectrical impedance analysis; DXA, dual-energy X-ray absorptiometry; CT, computed tomography; MRI, magnetic resonance imaging; SAT, subcutaneous adipose tissue; VAT, visceral adipose tissue; FM, fat mass; FFM, fat-free mass; LST, lean soft tissue; CVD, cardiovascular disease; 3DO, three-dimensional optical imaging; Se, sensitivity; Sp, specificity; CV, coefficient of variation; SEE, standard error of estimate; 4C, four-compartment model; ICC, intraclass correlation coefficient; CCC, concordance correlation coefficient.

## Data Availability

No new data were created or analyzed in this study. Data sharing is not applicable to this article.
